# Supplementary material for: The Anti-Nucleocapsid IgG Antibody as a Marker of SARS-CoV-2 Infection for Hemodialysis Patients
Source: Vaccines (Basel). 2025 Jul 13;13(7):750. doi: 10.3390/vaccines13070750 (PMC12299975; doi:10.3390/vaccines13070750)
Supplement: Supplementary file 1 [file vaccines-13-00750-s001.zip › vaccines-3688331-supplementary.pdf]

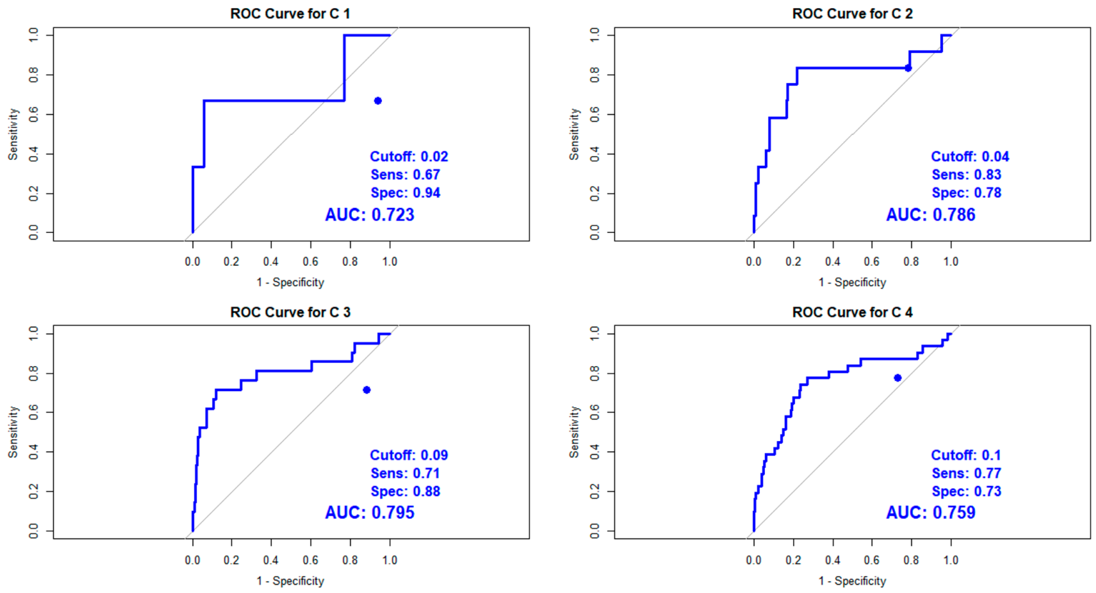

Supplementary Figure S1. Receiver Operating Characteristic Curves for SARS-CoV-2 Anti-Nucleocapsid IgG Antibody Testing in Hemodialysis Patients at Four Sampling Time Points, Adjusted for Days Since Last Vaccine, Number of Vaccines, Age, and BMI.
